# Food Insecurity Among Australian University Students Is Higher and More Severe Across an Extended Period of High Inflation: A Repeated Cross‐Sectional Study 2022–2024

**DOI:** 10.1002/hpja.70037

**Published:** 2025-04-07

**Authors:** Katherine Kent, Denis Visentin, Corey Peterson, Catherine Elliott, Carmen Primo, Sandra Murray

**Affiliations:** ^1^ School of Medical Indigenous and Health Sciences University of Wollongong Wollongong New South Wales Australia; ^2^ School of Health Sciences University of Tasmania Launceston Tasmania Australia; ^3^ Sustainability Unit University of Tasmania Launceston Tasmania Australia

**Keywords:** Australia, food insecurity, university student, zero hunger

## Abstract

**Introduction:**

Increasing financial pressures, resulting from a period of high inflation in 2022 and sustained into 2024, may have exacerbated food insecurity among Australian university students. This study aimed to determine the change in prevalence and severity of food insecurity among Australian university students between 2022 and 2024.

**Methods:**

Repeated cross‐sectional, online surveys measured food insecurity using the United States Department of Agriculture Household Food Security Survey Module six‐item short form (USDA HFSSM) in addition to six demographic and education characteristics. Students were categorised as being food secure or being marginally, moderately, or severely food insecure. Using a binary variable (food secure vs. food insecure), multivariate logistic regression identified students at higher risk of food insecurity. Independent proportions and logistic regression, adjusting for relevant predictors of food insecurity, measured change in the prevalence of food insecurity between 2022 and 2024.

**Results:**

In 2022 (*n* = 1249 students) and 2024 (*n* = 1603), younger, on campus, and international students experienced significantly higher odds of food insecurity. Marginal and moderate food insecurity were unchanged between 2022 and 2024. Severe food insecurity increased from 17% in 2022 to 29% in 2024 (95% CI −0.07, −0.13, *p* < 0.001), contributing to an overall significant increase in total food insecurity from 42% to 53% (Adjusted Odds Ratio: 1.6; 95% CI: 1.3, 1.8; *p* < 0.001).

**Conclusion:**

This study has identified a high prevalence and worsening severity of food insecurity among Australian university students during a period of high and sustained inflation.

**So What?:**

There is a need for immediate action, including health promotion initiatives and policies to uphold Australian university students' right to food.

## 1 Introduction

In line with international research [1], a growing body of evidence in the Australian context has reported that large proportions of university students experience food insecurity, which is defined as inconsistent access to enough safe and nutritious food to meet an individual's dietary needs and food preferences for an active, healthy life [2]. Australian studies have reported food insecurity prevalence statistics ranging from 13% to 54% [3, 4, 5, 6, 7, 8, 9, 10, 11, 12, 13, 14, 15], highlighting the extent of this issue across different university settings. Australian university students are at higher risk of food insecurity compared to the general population due to various intersecting factors. For example, university students typically face financial strain from high tuition fees, high housing costs, and the challenges of accessing affordable, healthy food options, particularly while studying on campus [16]. The groups of students at higher risk of food insecurity in these studies include students of younger age, who may be less experienced in budgeting and meal planning; students on low incomes and those receiving government support payments, which might be insufficient to cover all living expenses [4, 5]; students living out of home or on campus due to additional costs for accommodation and who may lack access to family support for meals [5, 6]; and international students [7, 12] who often face higher tuition fees, limited work opportunities and may struggle to afford or access culturally appropriate foods [17].

Food insecurity among university students is strongly associated with poorer mental health outcomes. Several Australian studies have found that students experiencing food insecurity report significantly higher levels of psychological distress, anxiety, and depression. For example, one study conducted in late 2020 reported that 42% of university students experienced food insecurity, and food insecurity was associated with significantly higher odds of psychological distress [3]. In line with this, another study from 2020 reported 48% of university students experienced food insecurity, which was associated with an increase in depressive symptoms [12]. Similarly, 32% of university students were reported to experience food insecurity in a study conducted during 2020 COVID‐19 associated lockdowns, and this was associated with higher odds of depression and anxiety in this sample [13]. Another study reported that the students experiencing food insecurity have a higher risk of psychological distress, which significantly worsens their overall academic performance [18]. Beyond mental health, food insecurity is linked to poorer diet quality, which can have health consequences for university students in both the short and longer term. A 2022 study reported more than half (54%) of Australian university students experienced food insecurity, and this was associated with significantly poorer diet quality and two‐times higher odds of using the on‐campus food pantry [11]. Aligning with this, a study conducted in late 2022 reported that 46% of university students experienced moderate and severe food insecurity, and that few students consumed adequate fruit and vegetables [14]. While a lower food insecurity prevalence statistic was reported by another study conducted in 2022 (19% for domestic students and 13% for international students), the negative impacts on diet quality persisted [15]. The reasons for food insecurity impacting diet quality have been studied in university students, identifying that food pricing, food availability, and food knowledge were major barriers to accessing healthy foods [16], and that being both time and energy poor as university students impacted negatively on their eating habits [16]. However, students with higher cooking skills and cooking frequency tend to have better dietary outcomes, suggesting that food literacy may be a protective factor [10].

Interestingly, food insecurity estimates among university students appear to be worsening in the past few years, where most of the studies reporting the higher range of prevalence statistics have been conducted since 2020 onwards. This suggests that economic pressures may be worsening, exacerbated by inflation, rising living costs, and the COVID‐19 pandemic. Since early 2022, when the last published statistics of food insecurity in Australia were collected, social and environmental challenges stemming from the COVID‐19 pandemic, ongoing global conflicts, natural disasters, and importantly, growing inflation have likely disproportionately impacted the financial wellbeing of university students. These challenges have not only affected their financial stability but also their fundamental human right to adequate food [19]. The period between 2022 and 2024 saw significant global economic challenges, including high inflation rates and rising costs of living. Inflation has both intensified and persisted [20]. Inflation unexpectedly surged from 2.1% in late 2021 to 8.4% in late 2022, driven by a culmination of factors such as supply disruptions, growing demand for commodities, the Russian invasion of Ukraine, and claims of corporate price‐gouging including for food. This was the highest inflation rate in over three decades and disproportionately has impacted the cost of food, in particular the cost of healthy food in Australia [21, 22]. Over the 12 months to the June 2024 quarter, the consumer price index (CPI) rose 3.8%, indicating that while inflation had somewhat eased compared to 2022, the persistence of high inflationary pressures remained during this time [23]. Consequently, financial demands from the growing cost‐of‐living, including for housing [24] and other university‐related factors like placement poverty [25] may have compounded university students' difficulties in meeting their basic needs. This underscores the need for further research into food insecurity among university students in Australia during this period of sustained inflation. Current statistics on food insecurity rates, allowing for comparison with previous years, could be used to track trends and assess whether the situation has worsened due to recent economic pressures. Such statistics, including the demographic groups at higher risk, could help advocacy towards universities and policymakers to develop more effective, targeted interventions to support vulnerable students and ensure their right to adequate food is upheld. Therefore, the aim of this study was to assess and compare the prevalence and severity of food insecurity among university students in Australia in 2022 and 2024, a period of high and sustained inflation, and identify the demographic predictors of food insecurity at both time points.

## 2 Methods

### 2.1 Study Setting and Participants

With a student population of approximately 29 259 students, University of Tasmania (UTAS) is a predominantly regional university that operates with three main campuses in Tasmania, Australia's only island state, and one smaller campus in Sydney. The university's predominantly regional setting provides unique opportunities for research and education, particularly in areas related to sustainability, environmental science and rural health. The UTAS Strategic Framework for Sustainability aims to position the university as a leader in sustainability. To support this, a biennial University Sustainability Survey is conducted, with the first survey conducted in 2016. The surveys aim to gather data on staff and students' sustainability perceptions, aspirations and behaviours, and the results are used to inform sustainability strategies and initiatives. The topics of food and food insecurity have been included as relevant to the survey since they directly impact the well‐being and sustainability practices of the university community. Addressing these issues supports the university's broader sustainability goals by ensuring access to sustainable and secure food systems on campus. Therefore, food insecurity has been previously measured in the 2020 and 2022 sustainability surveys [7, 9].

### 2.2 Questionnaire

In both the 2022 and 2024 surveys, the 6‐item United States Department of Agriculture Household Food Security Survey Module (USDA HFSSM) was employed to evaluate food insecurity among students [26]. This validated tool asks six questions about food access and consumption issues due to financial constraints [27]. Validation studies show that against the full 18‐item HFSSM, the 6‐item demonstrates high sensitivity (98%) and specificity (92%) with good reliability (*α* = 0.87) [26, 27, 28]. Affirmative responses to each of the six questions were assigned a score of 1, with total scores indicating the level of food insecurity [29]: food secure (0), marginally (1), moderately (2–4) or severely food insecure (5, 6). A binary variable was also created with students classified as food secure (0) or food insecure (1+). Marginal food insecurity was classified as food insecure in this study to align with international recommendations and recent research indicating that marginal food security can impact students similarly to higher levels of food insecurity [30]. The survey also gathered data on student demographics, including age, gender, study level (first year, second year, third year or more), degree type (pre‐degree, undergraduate or postgraduate), study mode (distance or on campus) and enrolment status (international or domestic). The study was conducted in accordance with the Declaration of Helsinki, and approval for these surveys was granted by the UTAS Human Research Ethics Committee (Reference number H0015525).

### 2.3 Data Collection

All UTAS students enrolled at the time were invited to participate via email to complete an online survey. Beyond being an enrolled student at UTAS, there was no other specific inclusion or exclusion criteria. The 2022 survey was emailed to ~31 000 students, and data collection took place from 7 March 2022 to 20 March 2022. The 2024 survey was emailed to ~34 000 students, and data collection took place from 14 March 2024 to 28 March 2024. Recruitment also involved promoting the surveys through newsletters and social media channels. Both surveys were hosted on the Qualtrics online survey platform. Participation was anonymous and voluntary. Participants were provided with a participant information sheet on the first page of the survey. Consent was implied by proceeding from the information page to the survey itself, as explained in the participant information sheet.

### 2.4 Data Analysis

Statistical analysis was conducted using IBM SPSS Statistics for Windows, version 26.0 (IBM Corp. Armonk, NY, USA). Descriptive statistics were calculated to summarise the characteristics of the survey respondents at both timepoints (2022 and 2024). The variables included age, gender, year of enrolment, degree level, mode of study and international student status. To assess differences in food insecurity prevalence between 2022 and 2024, independent samples comparisons were conducted. Proportions tests were used to compare total food insecurity, as well as the severity categories (marginal, moderate and severe food insecurity). To evaluate the change in food insecurity from 2022 to 2024 adjusting for relevant covariates, multivariate logistic regression models were used. The primary outcome variables were total food insecurity and the severity categories (marginal, moderate and severe food insecurity), with the year of survey as the key predictor. Demographic and academic covariates were included in the model if they were predictors of food insecurity in either 2022 or 2024 (*p* < 0.05) and therefore age, gender, year of enrolment, degree of enrollment, mode of study and international student status were included as covariates. To identify the demographic groups at risk in 2022 and 2024, binary logistic regression models were fitted separately for the 2022 and 2024 data to examine the odds of food insecurity based on demographic and academic characteristics. The models included age, gender, year of enrolment, degree level, mode of study and international student status as predictors. The regression model assumptions were assessed using Durbin–Watson statistics (to check for independence of observations), variance inflation factors (VIF; to determine collinearity with VIF statistic < 5). Data for all variables included in the regression models demonstrated equal variance, homoscedastic characteristics, and no significant outliers (partial regression plots, distributions of standardised residuals, histograms and P–P plots). Regression models were found to have satisfied all model assumptions. The significance level for all analyses was set at *p* ≤ 0.05.

## 3 Results

The survey sample comprised *n* = 1249 students in 2022 and *n* = 1603 in 2024. As described in Table 1, when comparing the respondents from both timepoints, the samples were broadly similar in demographic variables assessed. There was a very similar proportion across age groups, with younger respondents aged 18–24 years (44.2%, *n* = 705 in 2024 compared to 42.8%, *n* = 531 in 2022) the most common in each sample. Gender distribution also remained relatively consistent, with females comprising more than two‐thirds of the samples (68.0% (*n* = 1089) in 2024 and 68.9% (*n* = 861) in 2022). In terms of years of enrolment, more than half the participants in 2024 were first‐year students (52.0%; *n* = 834), which was higher compared to 42.0% (*n* = 525) in 2022. The proportion of students in pre‐degrees, undergraduate, and postgraduate programs was similar (Table 1), as was their mode of study (on campus students 56.8% (*n* = 709) in 2022 to 58.5% (*n* = 937) 2024). The proportion of international students increased slightly from 15.5% (*n* = 193) in 2022 to 17.8% (*n* = 286) in 2024.

**TABLE 1:** Demographic and education characteristic of the study sample, and proportion of each demographic level experiencing marginal, moderate and severe food insecurity in 2022 and 2024.

		Total *n* (%)				Total food insecure		Marginally food insecure		Moderately food insecure		Severely food insecure	
		2022		2024		2022	2024	2022	2024	2022	2024	2022	2024
Total		*N* = 1249		*N* = 1603		41.6%	52.7%	8.2%	8.2%	16.6%	17.7%	16.7%	26.8%
Age in years	18–24	531	(42.8%)	705	(44.2%)	48.0%	58.7%	10.2%	9.4%	19.2%	19.3%	18.6%	30.1%
	25–34	328	(26.4%)	410	(25.7%)	47.0%	59.5%	8.2%	8.5%	15.9%	19.5%	22.9%	31.5%
	35–44	174	(14.0%)	234	(14.7%)	36.8%	50.0%	5.2%	7.3%	18.4%	17.9%	13.2%	24.8%
	45–54	109	(8.8%)	135	(8.5%)	24.8%	34.1%	6.4%	6.7%	11.0%	11.1%	7.3%	16.3%
	55+	99	(8.0%)	110	(6.9%)	16.2%	20.0%	5.1%	4.5%	8.1%	9.1%	3.0%	6.4%
Gender	Male	346	(27.7%)	449	(28.0%)	43.6%	53.2%	8.4%	8.5%	17.9%	19.4%	17.3%	25.4%
	Female	861	(68.9%)	1089	(68.0%)	39.4%	51.9%	7.8%	8.3%	15.9%	16.8%	15.7%	26.8%
	Non‐binary	42	(3.4%)	65	(4.0%)	69.0%	63.1%	16.7%	6.2%	19.0%	20.0%	33.3%	36.9%
Years of enrolment	First year	525	(42.0%)	834	(52.0%)	45.7%	53.1%	8.2%	9.1%	19.0%	16.1%	18.5%	27.9%
	Second year	309	(24.7%)	322	(20.1%)	40.1%	54.0%	8.7%	6.8%	16.8%	20.2%	14.6%	27.0%
	Third year or longer	415	(33.2%)	447	(27.9%)	37.3%	54.0%	8.0%	7.6%	13.3%	21.3%	16.1%	25.1%
Degree of enrolment	Pre‐degree or short course	364	(29.1%)	501	(31.8%)	38.5%	48.5%	7.7%	6.6%	14.0%	14.8%	16.8%	27.1%
	Undergraduate (inc. honours)	567	(45.4%)	664	(42.1%)	44.4%	57.7%	9.9%	9.9%	18.5%	18.7%	16.0%	29.1%
	Postgraduate	318	(25.5%)	412	(26.1%)	39.9%	49.5%	6.0%	7.8%	16.0%	19.9%	17.9%	21.8%
Mode of study	On campus	709	(56.8%)	937	(58.5%)	48.0%	60.5%	9.6%	9.1%	18.9%	20.4%	19.5%	31.1%
	Distance	540	(43.2%)	666	(41.5%)	33.1%	41.7%	6.5%	7.1%	13.5%	13.8%	13.1%	20.9%
Enrolment status	Domestic	1056	(84.5%)	1317	(82.2%)	38.1%	50.6%	7.5%	8.2%	15.8%	17.0%	14.8%	25.4%
	International	193	(15.5%)	286	(17.8%)	60.6%	62.2%	12.4%	8.4%	20.7%	20.6%	27.5%	33.2%

### 3.1 Change in Food Insecurity Between 2022 and 2024

**FIGURE 1:**
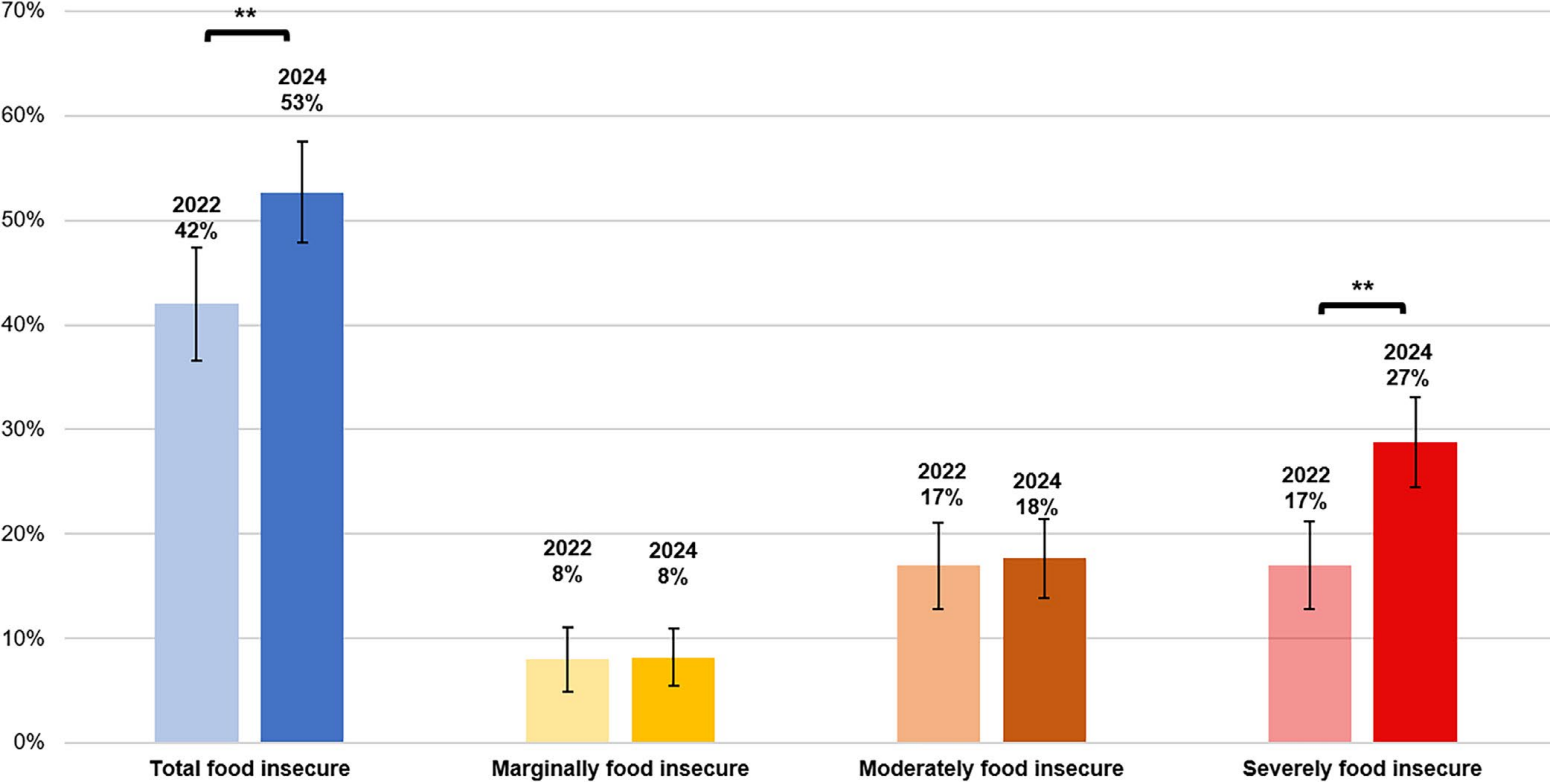
Prevalence and severity of food insecurity among university student survey respondents in 2022 (*n* = 1249) and 2024 (*n* = 1603); *p* value derived from independent samples proportions test: ** = *p* < 0.001.

Our surveys indicate a marked increase in total food insecurity from 42.0% (*n* = 524) in 2022 to 52.7% (*n* = 850) in 2024 (Figure 1). An independent‐samples proportions test showed that the increase of 11.2% (95% CI: [7.5%, 14.8%]; Table 2) was statistically significant. In terms of severity, marginal food insecurity remained unchanged (8.2% (*n* = 103) in 2022 and 8.2% (*n* = 131) in 2024) for the total sample (Figure 1), and moderate food insecurity was slightly higher from 16.6% (*n* = 207) in 2022 to 17.7% (*n* = 284) in 2024 (Figure 1), but an independent‐samples proportions test showed no significant difference (Table 2). Severe food insecurity, however, increased from 16.7% (*n* = 212) in 2022 to 26.8% (*n* = 465) in 2024 (Figure 1). An independent‐samples proportions test revealed this was a statistically significant increase of 10.1% (95% CI: [7.1%, 13.1%], Table 2).

**TABLE 2:** Independent samples proportion test and multivariate logistic regression of the changes in total food insecurity, and marginal, moderate and Severe Food Insecurity Among Students between 2022 and 2024.

Food insecurity category	Independent samples proportion test				Multivariate logistic regression			
	Proportion change %	95% CI	Wald Z	*p*	Adjusted odds ratio (AOR)*	Standard error (SE)	95% confidence interval (CI)	*p*
Total food insecurity	11.2%	[7.5%,14.8%]	−5.92	< 0.001	1.56^a^	0.079	[1.33, 1.82]	< 0.001
Marginal food insecurity	0%	[−2.1%, 2.0%]	0.01	0.991	0.994^b^	0.139	[0.757, 1.305]	0.966
Moderate food insecurity	1.1%	[−1.7%,3.9%]	−0.76	0.448	1.078^c^	0.102	[0.883, 1.316]	0.460
Severe food insecurity	10.1%	[7.1%,13.1%]	−6.41	< 0.001	1.77^d^	0.097	[1.47, 2.14]	< 0.001

* Age, gender, year of enrolment, degree of enrolment, mode of study, and international student status included as covariates.

^a^ Pseudo *R* ^2^ = 0.055 and a Likelihood ratio test statistic of χ^2^ = 1628.9, *p* < 0.0001.

^b^ Pseudo *R* ^2^ = 0.005, and the Likelihood ratio test statistic was χ^2^ = 12.958, *p* = 0.073.

^c^ Pseudo *R* ^2^ = 0.010, and the Likelihood ratio test statistic was χ^2^ = 28.363, *p* < 0.001.

^d^ Pseudo *R* ^2^ = 0.043 and a Likelihood ratio test statistic of χ^2^ = 124.445, *p* < 0.0001.

Multivariate logistic regression, adjusted for all demographic and education variables, showed a significant increase in the odds of total food insecurity in 2024 compared to 2022 (Table 2), indicating greater than 50% higher odds of food insecurity among students compared to 2022, after adjusting for all other factors. The odds of experiencing marginal and moderate food insecurity were not significantly different in 2024 compared to 2022 after adjusting for all other factors. However, the odds of severe food insecurity are also significantly higher in 2024, indicating 77% higher odds of severe food insecurity among students compared to 2022, after adjusting for all other factors (Table 2).

### 3.2 Groups of Students at Higher Odds of Food Insecurity

Food insecurity increased across all age groups from 2022 to 2024, with the biggest increase reported among the younger age groups: 18–24 years (48.0% (*n* = 255) in 2022 to 58.7% (*n* = 414) in 2024) and 25–34 years (47.0% (*n* = 154) in 2022 to 59.5% (*n* = 244) in 2024). Students in the 55+ age group had the smallest increase, from 16.2% (*n* = 16) in 2022 to 20.0% (*n* = 22) in 2024. In 2022, the 18–24 age group had 4.8 times higher odds of food insecurity compared to those aged 55+, which rose to 5.7 times higher in 2024 (Table 3). Similarly, the 25–34 group saw an increase from 4.6 times higher odds in 2022 to 5.9 times higher in 2024. The risk for the 45–54 age group also increased from non‐statistically significant odds of 1.7 in 2022 to a significantly higher odds of 2.1 when compared to the 55+ group (Table 3).

**TABLE 3:** Univariate logistic regression results of food security with demographic characteristics for Australian university students in 2022 and 2024.

Characteristic		2022				2024				
		Odds ratio	SE	95% CI	*p*	Odds ratio	SE	95% CI		*p*
Age in years	18–24	4.8	0.3	[2.7, 8.4]	< 0.001	5.7	0.3	[3.5, 9.3]		< 0.001
	25–34	4.6	0.3	[2.6, 8.2]	< 0.001	5.9	0.3	[3.5, 9.8]		< 0.001
	35–44	3.0	0.3	[1.6, 5.6]	< 0.001	4.0	0.3	[2.3, 6.8]		< 0.001
	45–54	1.7	0.4	[0.9, 3.4]	0.128	2.1	0.3	[1.1, 3.7]		0.015
	55+	Reference category								
Gender	Man or male	Reference category								
	Woman or female	0.8	0.1	[0.7, 1.1]	0.172	0.9	0.1	[0.8, 1.2]		0.631
	Non‐binary, self‐identify or prefer not to disclose	2.9	0.4	[1.4, 5.7]	0.003	1.5	0.3	[0.9, 2.6]		0.138
Years of enrolment	First year	1.4	0.1	[1.1, 1.8]	0.010	1.1	0.1	[0.9, 1.4]		0.471
	Second year	1.1	0.2	[0.8, 1.5]	0.447	1.1	0.1	[0.8, 1.5]		0.407
	Third year or longer	Reference category								
Degree of enrolment	Pre‐degree or short course	Reference category				—	—	—		—
	Undergraduate (including honours)	1.3	0.1	[1.0, 1.7]	0.071	1.4	0.1	[1.1, 1.8]		0.002
	Postgraduate	1.1	0.2	[0.8, 1.4]	0.694	1.0	0.1	[0.8, 1.4]		0.761
Mode of study	On campus	1.9	0.1	[1.5, 2.3]	< 0.001	2.1	0.1	[1.7, 2.6]		< 0.001
	Distance	Reference category			—					
Enrolment status	Domestic	Reference category				—	—	—		—
	International	2.5	0.2	[1.8, 3.4]	< 0.001	1.6	0.1	[1.2, 2.1]		< 0.001

*Note:* The 2022 binary logistic regression results have been previously reported [9].

Males and females both experienced increases in total food insecurity, which increased by 9.6% for males (43.6% (*n* = 151) in 2022 to 53.2% (*n* = 239) in 2024) and 12.5% for females (39.4% (*n* = 339) in 2022 to 51.9% (*n* = 565) in 2024) (Table 1). Males and females did not have significantly different odds of food insecurity in either 2022 or 2024. Those identifying as non‐binary had reported a decrease in total food insecurity (69.0% (*n* = 29) in 2022 to 63.1% (*n* = 41) in 2024). While this group had significantly higher odds of food insecurity in 2022 (OR = 2.9), compared to males, this reduced to 1.5 in 2024 but was not found to be significantly higher (Table 3).

First‐year students experienced an increase in food insecurity from 45.7% (*n* = 240) in 2022 to 53.1% (*n* = 443) in 2024. While they had higher odds of being food insecure in 2022, they no longer had statistically significantly higher odds compared to third‐year students in 2024 (Table 3). Despite the rise in prevalence for first‐year students, the food insecurity rates for third‐year or longer students increased more, narrowing the relative difference. Similarly, second‐year students saw a rise from 40.1% (*n* = 124) in 2022 to 54.0% (*n* = 174) in 2024, but their odds of food insecurity were also not statistically higher than third‐year students.

Food insecurity increased across all degree types, with undergraduate students showing the largest increase from 44.4% (*n* = 252) in 2022 to 57.7% (*n* = 383) in 2024. Therefore, when compared to pre‐degree and short course students, the odds of being food insecure for undergraduate students went from being not statistically significant in 2022 to statistically significantly higher in 2024 (Table 3). Both on campus and distance students experienced increases in food insecurity prevalence, with on campus students showing a larger increase (from 48.0% (*n* = 341) in 2022 to 60.5% (*n* = 567) in 2024) compared to distance students (from 33.1% (*n* = 179) in 2022 to 41.7% (*n* = 278) in 2024). While at higher risk at both time points, the odds of being food insecure for on campus students compared to distance students increased slightly from OR 1.9 in 2022 to OR 2.1 in 2024.

Both domestic and international students reported increases in food insecurity prevalence, with domestic students experiencing a larger increase (from 38.1% (*n* = 402) in 2022 to 50.6% (*n* = 667) in 2024) compared to international students (from 60.6% (*n* = 117) in 2022 to 62.2% (*n* = 178) in 2024; Table 1). Therefore, the odds of being food insecure for international students compared to domestic students decreased from OR 2.5 in 2022 to OR 1.6 in 2024 (Table 3), suggesting a narrowing of the gap between these two groups.

## 4 Discussion

This study explored how the prevalence and severity of food insecurity changed for Australian university students between 2022 and 2024, during a period of high, sustained inflation. Our findings suggest a significant increase in food insecurity among university students. Most concerningly, there has been a substantial rise in severe food insecurity, where students are skipping meals and experiencing hunger, indicating a potentially growing crisis in student welfare. Our findings have significant implications as they suggest that an increasing number of students are facing financial challenges that could negatively impact their academic performance, physical and mental well‐being and this could be potentially contributing to higher dropout rates from university.

In our study, the proportion of students experiencing food insecurity in 2024 appears to have increased to more than half of students, and to the best of our knowledge, this is the highest food insecurity prevalence statistic reported in studies of Australian university students in any setting. Of concern is the relative stability of marginal and moderate food insecurity, but the significant increase in severe food insecurity to nearly a third of student respondents in 2024. These results align with some broader trends observed in studies on food insecurity during periods of inflation in broader populations [31]. For example, a study of 1284 adults, conducted in the same region as our study, reported that food insecurity impacted 39% of survey respondents in 2022 when food inflation was high. The study also found that food‐insecure respondents used a variety of coping strategies beyond just cutting meals, as they were more likely to rely on credit for living expenses, reduce housing costs and cut discretionary spending, such as doctor and dentist visits, to manage their finances while experiencing food insecurity [31]. Our study builds on this evidence by showing that university students are particularly vulnerable to food insecurity during periods of inflation. In fact, other evidence suggests that on average, the proportion of university students experiencing food insecurity is four times higher than in general populations [32]. These findings underscore the need for targeted interventions to address food insecurity during periods of inflation both in the general population and among university students, specifically.

The comparative analysis of food insecurity at two time points in our study allows for a deeper understanding of the changing patterns among university students. Most Australian studies that measure the prevalence of food insecurity are cross‐sectional in nature [33], and therefore, report food insecurity at one point in time. However, some more recent studies internationally comparing food insecurity before and during the COVID‐19 pandemic have reported an upward trend in food insecurity among college students. For example, food insecurity worsened for 20% of students attending a large public university in Southeastern USA [34]. Similarly, around 22% of college students in another American study reported that the COVID‐19 pandemic worsened their food insecurity [35]. Students in both studies attributed these increases to changing employment status and other public health restrictions that reduced physical and economic access to food. However, the COVID‐19 pandemic restrictions had ended by 2022 in Australia, and so similar factors related to COVID‐19 are unlikely to fully explain the escalation of food insecurity identified in our study.

The observed increase in food insecurity among university students in our study occurred during a period of sustained inflation and broader economic pressures, which have been widely reported in Australia. While our study did not directly assess the influence of these factors, previous research suggests that economic conditions can impact food security at a population level [31]. These factors have been described by the Australian Government and more broadly as a ‘*cost of living crisis*’ [36]. Similar challenges have faced other countries during this period [37]. These macroeconomic factors could have had a disproportionate impact on university students, who often have limited income and financial reserves [38]. One study has reported that the cost of food alone has increased by 8% between 2022 and 2024 [39]. Further, housing costs in Australia have escalated. While our study did not directly assess the impact of housing stress on student food insecurity, prior research has identified this a potential contributing factor to food insecurity [40]. In particular, the Australian rental market has experienced strong demand and slowing supply, which has contributed to a deterioration in rental affordability and an increase in financial stress for some renter households [41], including for university students who may already struggle to cover basic living expenses. With a significant portion of their income diverted towards housing costs [42], university students may be left with little money for essentials like food, but this requires further research. Indeed, some research has reported that when international university students in Australia had financial issues, they were less inclined to spend money on food and other essentials like medical services [43]. Furthermore, university degrees are becoming more expensive. In 2024, Australian university students will face an average 7.8% increase in their tuition fees, which may impact food insecurity in both the short and longer‐term [44]. Many domestic students may be eligible for government loans that help cover these costs in the short‐term, however, impact on students in the longer term may vary depending on a student's individual financial circumstances and ability to make the loan repayments after graduating. Future research could examine whether rising tuition fees contribute to financial strain affecting students' ability to afford food.

Examining the demographic changes in our study, we observe that younger students, particularly those aged 18–24 and 25–34, experienced the most substantial increases in food insecurity between 2022 and 2024. This heightened vulnerability of younger students may be attributed to their more limited financial resources and less established support networks when compared to middle‐aged students [45]. Further, our study identified a persistently higher risk of food insecurity for on‐campus enrolled students compared to distance learners. This may indicate that existing resources provided by universities for food‐insecure students are under increasing strain. Both government financial aid and university on‐campus support services, including food assistance programmes, may now be insufficient to meet the growing demand among university students, warranting further research. Interestingly, our study shows that international students experienced higher odds of food insecurity compared to domestic students, but by 2024, this disparity lessened (although was still evident), as the proportion of food‐insecure domestic students increased. This shift might be due to several factors. During the COVID‐19 pandemic, targeted government financial support measures were available for domestic students, but not international students, leaving international students at higher risk of food insecurity. This prompted the initiation of other emergency relief funds and food assistance programmes for international students. Between 2022 and 2024, it is possible that domestic students may have had reduced access to support as the pandemic‐related measures receded, making the difference between the two groups less pronounced. However, further investigation of this is needed. Future research should examine the specific factors contributing to the increased food insecurity among all the groups of university students at highest risk of food insecurity and evaluate the effectiveness of current support systems in meeting their needs.

Addressing these intersecting challenges requires a rights‐based approach that recognises adequate food as a fundamental human right [19] and calls for targeted interventions at the student level, university level and national level to ensure that all students have access to sufficient, safe and nutritious food. At the student level, targeted interventions for the most vulnerable groups identified in our study, such as younger students and those living on campus, are important. For example, financial literacy programs could empower students to manage their finances more effectively and could reduce the risk of food insecurity [46]. Additionally, food literacy programs that teach cooking skills, meal preparation and grocery budgeting have been linked to better diet quality and food security among university students [47]. Finally, peer‐led initiatives and student‐run food cooperatives can foster a sense of community while providing cost‐effective ways to access fresh and affordable food [48].

Universities must also urgently reassess and enhance their support systems for students at an organisational level. When food relief initiatives are provided by universities, some Australian evidence shows that food insecure university students do access these services [11, 12]. Sometimes these services provide less healthy food options to students [16], which may be associated with the poor diet quality experienced by food insecure students, even when they are accessing campus food pantries [11]. While students tend to view these provisions favourably [49], food relief initiatives can be under‐resourced, irregular or difficult for students to access [16] leading to a situation where students, already grappling with financial stress, may find themselves unable to meet their basic nutritional needs. Such initiatives also tend to be under‐evaluated [50]. Research suggests that other approaches that maximise student agency in food procurement are important, including subsidised meal initiatives, such as low‐cost meal plans, university‐sponsored food and recipe boxes or supermarket vouchers [51, 52]. In addition to these short‐term strategies, universities should also implement policies and governance structures to support food outlets to provide affordable, healthy foods on campus [53]. Increasing emergency financial assistance for other basic needs beyond food, such as housing and transport, is also paramount for maintaining food security [54, 55].

Broader societal and policy responses are also essential, as individual efforts and university‐level interventions alone cannot fully address the structural drivers of food insecurity. While financial literacy programs, food relief initiatives and campus‐based meal subsidies provide important short‐term support, real and sustained improvements in student food security require systemic changes in social and economic policies. This may involve advocating for increased government funding for higher education, and prioritising policies that address the rising cost of living, particularly related to food. By adopting a ‘rights‐based’ approach, these measures can help ensure that all students have access to sufficient, safe and nutritious food [19]. Positively, the Australian government through the Australian Universities Accord 2024 report [56], has recognised the financial crisis of university students when undertaking long and unpaid professional placements, and has provided some funding to students within some disciplines, to alleviate some financial burden for students during this critical time. However, these measures may have not yet had a widespread impact and may not be sufficient to address the broader and more pervasive issue of food insecurity among university students throughout the rest of their degree.

To our knowledge, our study is the first to compare how the prevalence and severity of food insecurity has changed in an Australian university context at two time points, and a strength of our analyses is the large sample size for both surveys. Our study also has several limitations. Firstly, the cross‐sectional nature of our surveys limits our ability to track changes in individual students over time. This means we cannot determine causality or individual trajectories of food insecurity. Secondly, while we adjusted for various demographic and education factors, there may be other confounding variables not accounted for in our analysis, such as income, living situation, and family structure that could have impacted our results. Additionally, our sample, although large, may not be generalisable beyond the study sample. While participants in our study were not reimbursed for their time, the sustainability theme of the survey may have attracted students with a stronger interest in the topic, potentially biasing recruitment. In 2024, the sample size represented about 5.8% of enrolled students at UTAS. Our survey responses had a lower proportion of international students (18%, lower than the 21% in the overall student population), and pre‐degree students accounted for 31%, (significantly higher than the 14% in the broader UTAS student population). This suggests our findings may be skewed, limiting their generalisability. Further, these findings might not be generalisable to other settings and universities across Australia. For example, as a regional university in an island state, the students in our study may face unique challenges, such as higher food costs and limited food access, which differ from those at metropolitan universities on the mainland.

## 5 Conclusion

Our repeated cross‐sectional study found a significant increase in food insecurity among Australian university students between 2022 and 2024, coinciding with a period of sustained inflation. Severe food insecurity increased the most, with younger and on‐campus students being particularly affected. The gap in food insecurity between domestic and international students also narrowed. These findings highlight the need for strengthened support systems and targeted interventions in university settings. Universities and policymakers should consider strategies that address both immediate student needs and, in the longer term, address the broader socioeconomic factors influencing food insecurity.

## Author Contributions

Conceptualization, all authors. Methodology: all authors. Formal analysis: Katherine Kent and Denis Visentin. Resources: Corey Peterson. Writing – original draft preparation: Katherine Kent. Writing – review and editing: all authors. Project administration: Corey Peterson and Carmen Primo and Catherine Elliott. All authors have read and agreed to the published version of the manuscript.

## Consent

Informed consent was obtained from all subjects involved in the study.

## Conflicts of Interest

The authors declare no conflicts of interest.

## Data Availability

Data supporting this manuscript is not publicly available but can be made available upon reasonable written request to the corresponding author (K.K.).
